# Best practice guidance for antibiotic audit and feedback interventions in primary care: a modified Delphi study from the Joint Programming Initiative on Antimicrobial resistance: Primary Care Antibiotic Audit and Feedback Network (JPIAMR-PAAN)

**DOI:** 10.1186/s13756-023-01279-z

**Published:** 2023-07-29

**Authors:** Kevin L. Schwartz, Alice X. T. Xu, Sarah Alderson, Lars Bjerrum, Jamie Brehaut, Benjamin C. Brown, Heiner C. Bucher, An De Sutter, Nick Francis, Jeremy Grimshaw, Ronny Gunnarsson, Sigurd Hoye, Noah Ivers, Donna M. Lecky, Morten Lindbæk, Jeffrey A. Linder, Paul Little, Benedikte Olsen Michalsen, Denise O’Connor, Celine Pulcini, Pär-Daniel Sundvall, Pia Touboul Lundgren, Jan Y. Verbakel, Theo J. Verheij

**Affiliations:** 1 Public Health Ontario, 480 University Ave, Ste 300, Toronto, ON M5G 1V2 Canada; 2 Dalla Lana School of Public Health, University of Toronto, Toronto, Canada; 3 Unity Health Toronto, Toronto, Canada; 4 Leeds Institute of Health Sciences, University of Leeds, Leeds, UK; 5 Oaklands Health Centre, Holmfirth, UK; 6 Section of General Practice and Research Unit for General Practice, Department of Public Health, University of Copenhagen, Copenhagen, Denmark; 7 Centre for Practice-Changing Research (CPCR), Ottawa Hospital Research Institute, Ottawa, Canada; 8 Division of Population Health, Health Services Research and Primary Care, School of Health Sciences, University of Manchester, Manchester, UK; 9 Division of Clinical Epidemiology, University Hospital Basel and University of Basel, Basel, Switzerland; 10 Department of Public Health and Primary Care, Center for Family Medicine UGent, Ghent University, Ghent, Belgium; 11 Primary Care Research Centre, University of Southampton, Southampton, UK; 12 Department of Medicine, University of Ottawa, Ottawa, Canada; 13 General Practice/Family Medicine, School of Public Health and Community Medicine, Institute of Medicine, Sahlgrenska Academy, University of Gothenburg, Gothenburg, Sweden; 14 Research, Education, Development & Innovation, Primary Health Care, Region Västra Götaland, Gothenburg, Sweden; 15 Centre for Antibiotic Resistance Research (CARe) at University of Gothenburg, Gothenburg, Sweden; 16 Department of General Practice, Antibiotic Centre for Primary Care, Institute of Health and Society, University of Oslo, Oslo, Norway; 17 Women’s College Hospital, Toronto, Canada; 18 Primary Care and Interventions Unit, UK Health Security Agency, Gloucester, England; 19 Division of General Internal Medicine, Northwestern University Feinberg School of Medicine, Chicago, IL USA; 20 Primary Care Research Centre, University of Southampton, Southampton, England; 21 School of Public Health and Preventive Medicine, Monash University, Melbourne, Australia; 22 APEMAC, Université de Lorraine, Nancy, France; 23 CHRU-Nancy, Centre regional en antibiotherapie de la region Grand Est AntibioEst, Université de Lorraine, Nancy, France; 24 Department of Public Health, Nice University Hospital, Nice, France; 25 Department of Public Health and Primary Care, KU Leuven, Louvain, Belgium; 26 NIHR Community Healthcare Medtech and IVD Cooperative, Nuffield Department of Primary Care Health Sciences, University of Oxford, Oxford, UK; 27 Julius Center for Health Sciences and Primary Care, University Medical Center Utrecht, Utrecht, The Netherlands

## Abstract

**Background:**

Primary care is a critical partner for antimicrobial stewardship efforts given its high human antibiotic usage. Peer comparison audit and feedback (A&F) is often used to reduce inappropriate antibiotic prescribing. The design and implementation of A&F may impact its effectiveness. There are no best practice guidelines for peer comparison A&F in antibiotic prescribing in primary care.

**Objective:**

To develop best practice guidelines for peer comparison A&F for antibiotic prescribing in primary care in high income countries by leveraging international expertise via the Joint Programming Initiative on Antimicrobial Resistance—Primary Care Antibiotic Audit and Feedback Network.

**Methods:**

We used a modified Delphi process to achieve convergence of expert opinions on best practice statements for peer comparison A&F based on existing evidence and theory. Three rounds were performed, each with online surveys and virtual meetings to enable discussion and rating of each best practice statement. A five-point Likert scale was used to rate consensus with a median threshold score of 4 to indicate a consensus statement.

**Results:**

The final set of guidelines include 13 best practice statements in four categories: general considerations (n = 3), selecting feedback recipients (n = 1), data and indicator selection (n = 4), and feedback delivery (n = 5).

**Conclusion:**

We report an expert-derived best practice recommendations for designing and evaluating peer comparison A&F for antibiotic prescribing in primary care. These 13 statements can be used by A&F designers to optimize the impact of their quality improvement interventions, and improve antibiotic prescribing in primary care.

**Supplementary Information:**

The online version contains supplementary material available at 10.1186/s13756-023-01279-z.

## Introduction

Rising antimicrobial resistance (AMR) poses a threat for modern medicine and society as a whole. In 2019, an estimated five million global deaths were associated with bacterial AMR [1], and one of the pillars of the World Health Organization’s Global Action Plan to combat AMR is to optimize the use of antimicrobials in humans [2]. Global action is required to slow AMR to avoid a post-antibiotic era where serious bacterial infectious diseases can no longer be effectively treated. Misuse and overuse of antibiotics is an important contributor to this crisis. Furthermore, overuse of antibiotics also wastes resources, medicalizes minor illness, and harms patients by causing adverse effects such as increased antibiotic resistance, and disturbances in gut microbiome [3].

Primary care is responsible for 80–90% of human antibiotic usage, making general practitioners and pediatricians critical partner for antimicrobial stewardship [4, 5]. It is estimated that 25–50% of all antibiotics used in primary care settings in high income countries are unnecessary or inappropriate [6–9]. Effective antimicrobial stewardship programs should be multidisciplinary and include multifaceted evidence-based interventions that incorporate principles of behavioral science in order to effectively address the drivers of unnecessary and inappropriate antibiotic prescribing [5]. Audit and feedback (A&F) has been defined as “a summary of clinical performance of healthcare providers”, which has been used widely in healthcare to improve performance [10]. Interventions based on peer comparison A&F, which involves the comparison of clinical performance with other healthcare providers at individual or team level, are rooted in behavioral science, however their effectiveness may vary depending on how they are designed and implemented [10, 11]. A&F interventions have been trialed in a number of countries with variable formats, data sources, prescribing metrics, and effect sizes [12–16]. Best practice guidance for general audit and feedback exists [17, 18], though it is not specific to antibiotic prescribing or primary care, where other factors such as the selection of feedback recipients and outcome indicators should be taken into consideration. Peer comparison A&F is recognized as a potentially valuable intervention to improve antibiotic use in primary care [12–14, 19].

The Joint Programming Initiative on Antimicrobial Resistance—Primary Care Antibiotic Audit and Feedback Network (JPIAMR-PAAN) is an international network of over 40 members representing 15 countries with expertise in the fields of antimicrobial stewardship, primary care, and implementation science (Additional file 1: Table S1). The network was created through a funding call from JPIAMR. Invitations to participate in JPIAMR-PAAN were sent from network leads to experts in this field from Europe, Australia, and North America. Our objective was to extract and compile expert advice to develop best practice guidelines for peer comparison A&F of antibiotic prescribing in the primary care settings for high income countries.

## Methods

The Delphi technique aims to achieve a convergence of expert opinions via an iterative process of questionnaires and feedback. We used a modified Delphi process (MDP) with three rounds of surveys and feedback meetings to identify and develop best practice guidelines for peer comparison A&F of antibiotic prescribing in primary care settings of high-income countries [20–22].

### Modified Delphi process preparation and setting

This study presents results from expert consensus and did not require review or approval from an ethics review board. All members of JPIAMR-PAAN were intended panelists of the present MDP, with expertise in the fields of antimicrobial stewardship, primary care, and/or implementation science. JPIAMR-PAAN members represent 15 high-income countries in Australia, Europe, and North America (Additional file 1: Table S1). Network members provided verbal consent to participate in the MDP.

**Table 1:** Comparison of JPIAMR-PAAN’s 13 best practice recommendations on antibiotic audit and feedback interventions with Brehaut et al.’s 15 suggestions for practice feedback interventions

JPIAMR-PAAN 13 best practice recommendations on antibiotic audit and feedback interventions	Brehaut et al.[17] 15 recommendations for practice feedback interventions
*General considerations*	
1. Antibiotic audit and feedback interventions in primary care should be framed as quality improvement projects within a supportive environment	The specific framing of audit and feedback interventions was not considered in Brehaut’s original 15 suggestions
2. Prior to initiating an antibiotic audit and feedback intervention in primary care, consider potential barriers to success such as local data availability, data validity, expected engagement of feedback recipients, perceived patient expectations for antibiotics, and other situational factors	The Brehaut recommendations discuss potential barriers such as *“prevent defensive reactions to feedback”* – we discuss some potential barriers specific to antibiotic audit and feedback in primary care
3. Strategies to optimize reach and engagement of an antibiotic audit and feedback intervention in primary care include; utilizing an opt-out approach to delivery of feedback reports, offering of continuing medical education credits, financial incentives, and facilitated peer group discussions	The recommendation to *“Address barriers to feedback use”* is considered and we provide specific strategies to optimize engagement
*Selecting feedback recipients*	
4. All primary care prescribers, regardless of practice type or prescribing volume, should be included in antibiotic prescribing audit and feedback interventions	Recommendation for the selection of feedback recipients was not provided
*Data and indicator selection*	
5. Feedback indicators for antibiotic prescribing in primary care should target reductions in antibiotic initiations, prolonged antibiotic duration, and/or unnecessary broad-spectrum antibiotics	We built on the statement *“Recommend actions that are consistent with established goals and priorities”* and suggested specific indicators of high priority in antibiotic prescribing
6. Antibiotic feedback reports in primary care should enable and support behaviour change by providing guidance and educational resources	We agree with statements that *“Recommend actions that can improve and are under the recipient’s control”* and *“Recommend specific actions”*—such that we suggest antibiotic feedback reports to include specific action (e.g. reduce prescribing, prescribe for shorter duration) and evidence-based behaviour change messaging
7. The optimal data source for antibiotic audit and feedback in primary care is credible, valid, routinely collected, and comprehensive for the region; ideally containing prescription, diagnostic, and clinical data	No suggestion regarding the data sources for audits
8. Benchmarks or achievable targets for peer comparisons for antibiotic prescribing in primary care should be indicator specific and based on national and/or local performance data of high performing peers	We provide specific recommendations that support the statement *“Choose comparators that reinforce desired behavior change”* in the context of antibiotic prescribing
*Feedback delivery*	
9. Antibiotic audit and feedback in primary care should be displayed such that recipients can understand their performance and desired actions within seconds	We concur with many of the suggestions related to feedback display and delivery, including *“Closely link the visual display and summary message”*, *“Minimize extraneous cognitive load for feedback recipients”*, and *“Provide short, actionable messages followed by optional detail”* – all three statements will apply in the context of primary care antibiotic audit and feedback
10. Antibiotic audit and feedback reports in primary care should be repeated with updated data over time. The optimal frequency is not known but can depend on local factors such as data availability and seasonality of prescribing	We agree with the suggestion to *“Provide multiple instances of feedback”*. However we recognize the difficulty with the suggestion *“Provide feedback as soon as possible and at a frequency informed by the number of new patient cases”* in the context of antibiotic prescribing, as well as the paucity of data on the optimal frequency of feedback
11. Antibiotic feedback in primary care should be ideally delivered by multiple strategies including verbal, paper, and/or electronic means	We further support the recommendation to *“Provide feedback in more than one way”*
12. Antibiotic feedback should be delivered to primary care prescribers from a respected authority figure or colleague	We believe that feedback delivery from a respective authority figure or colleagues is an effective way to *“Address credibility of the information”*
13. Individual-level antibiotic feedback should be delivered confidentially to primary care prescribers, and the opportunity for peer discussion should be provided and encouraged	Within the context of antibiotic prescribing, we stress the significance to *“Provide individual rather than general data”* given the nature of prescribing. Furthermore, we strongly encourage peer discussion of individual feedback with other prescribers, as a way to facilitate the suggestion *“Construct feedback through social interaction”*

**Fig. 1:**
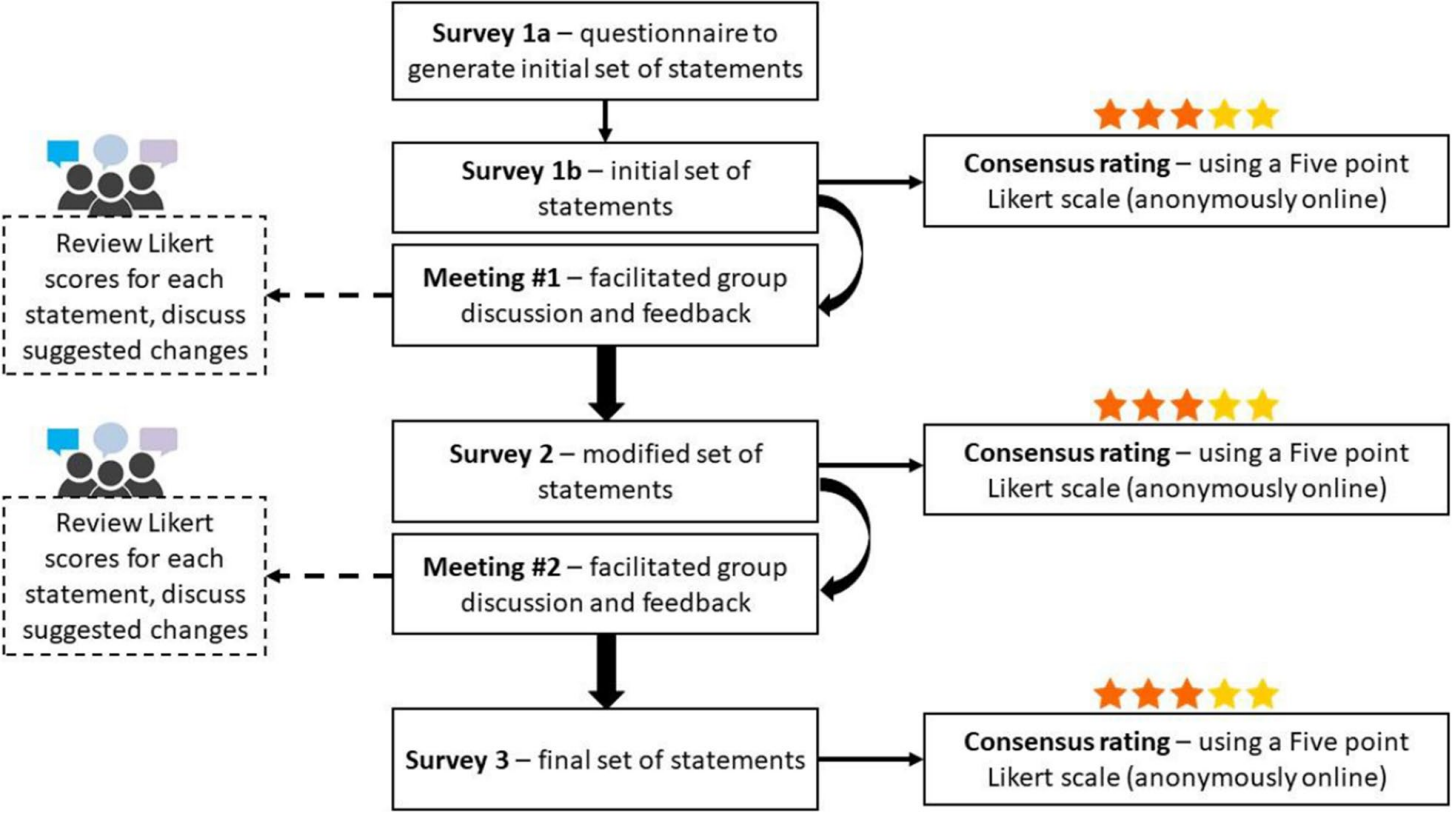
Modified Delphi Process

A five-point Likert scale (1-strongly disagree, 2-disagree, 3-neutral, 4-agree, and 5-strongly agree) was adopted as the consensus rating tool with the median threshold of percent agreement used for consensus [23, 24]. Frequency statistics and median scores were generated for each consensus statement. Statements that did not achieve consensus (median Likert score <  = 2.0) and borderline consensus statements (median Likert score > 2.0 but < 4.0) were discussed at length during feedback sessions; while statements that achieved consensus (median Likert score >  = 4.0) were not prioritized for discussion; however, all statements were reviewed and discussed during the meetings (Fig. 1).

Panelists of the MDP underwent three rounds of online surveys and consensus rating (Additional file 1: A), along with two virtual meetings or feedback sessions. All surveys were hosted on Nettskjema™, an online survey tool developed by the University Information Technology Center at University of Oslo. Both virtual meetings were hosted by virtual video conference on Zoom. Each round of the MDP was facilitated by JPIAMR-PAAN coordinators, who acted as process facilitators.

### Modified Delphi process round 1

Prior to Round 1 of the MDP, the core team of JPIAMR-PAAN, consisting of the five network leads (KS, NI, ML, SH, JG) and two coordinators (AX, BM), reviewed existing guidance on developing effective A&F interventions, and drafted an initial questionnaire with multiple choice and open-ended questions (Survey 1a). The core team drew on Brehaut et al.’s 15 suggestions for optimizing effectiveness [17], and incorporated the following topics in Survey 1a; General Statements on A&F, Desired action, Desired data, Feedback display, and A&F delivery.

Invitation to complete Survey 1a was emailed to all JPIAMR-PAAN members on March 28th, 2022. Members were invited to respond and/or suggest additions or modifications to the survey. Results were reviewed by the process facilitators, and an initial list of consensus statements was produced (Survey 1b). This initial list of best practice statements was sent by email to all JPIAMR-PAAN members for consensus rating and feedback on April 11^th^, 2022. Median Likert scores were obtained for each statement and subsequently used to facilitate the first virtual meeting.

### Modified Delphi process round 2

All JPIAMR-PAAN members were invited to attend the first virtual feedback session on April 21st, 2022. The median Likert score of each statement was presented by the process facilitators (AX and BM), followed by a facilitated discussion and clarification of feedback. Using suggestions and feedback generated from the virtual meeting, a modified set of statements was produced by the process facilitators (Survey 2). All network members were invited to complete Survey 2 for consensus rating and provide open-text feedback on May 30th, 2022.

### Modified Delphi process round 3

Following an iterative approach, the results from Survey 2 were used to facilitate discussion of the second virtual meeting on June 14th, 2022, with prioritization on statements that did not reach consensus. Feedback generated from the second meeting and Survey 2 were then used to produce the final set of statements (Survey 3). This final set of statements was distributed to all JPIAMR-PAAN members for a final round of consensus rating on July 11th 2022. All statements with a median Likert score >  = 4.0 were included in the final set of best practice recommendations.

## Results

We administered four online surveys and two virtual meetings in total as part of the MDP. All four surveys had a response rate of 54% (20 out of 37), with 12 panelists completing all four surveys. Meeting #1 and #2 had 14 and 13 panelists present, respectively; with eight panelists attending both meetings. Overall, seven panelists participated in all aspects of the MDP by responding to all surveys and attending both meetings. The final product includes four categories and 13 best practice recommendations for designing and evaluating A&F interventions for antibiotic prescribing in primary care. Table 1 presents the comparison between our 13 recommendations with Brehaut et al.’s 15 recommendations for practice feedback interventions.

### Best practice recommendations

The best practice recommendations below are presented with a summary of internal discussions and comments around each statement.

## General considerations

**Antibiotic audit and feedback interventions in primary care should be framed as quality improvement projects within a supportive environment.**

There exists substantial variability in antibiotic prescribing in primary care [25, 26]. A&F is a commonly employed tool to improve quality of care. The goal of A&F programs should be to support clinicians to improve through the presentation of their own data in relation to their peers and/or standard of care. Historically, poorly designed audits can result in clinicians feeling threatened, rather than supported, by top-down feedback [27]. This is further supported by the hypotheses of ‘function’ and ‘ownership’ from Clinical Performance Feedback Intervention Theory (CP-FIT); which posits that feedback interventions are more effective when clinicians are perceived to support positive change, and “own” the process rather than imposed upon [18]. Those designing A&F should consider the importance of framing their interventions as quality improvement, engage clinicians and patients in the process, and be mindful of their messaging to maintain the shared focus of improving the quality of patient care through optimizing the use of antibiotics.

**Prior to initiating an antibiotic audit and feedback intervention in primary care, consider potential barriers to success such as local data availability, data validity, expected engagement of feedback recipients, perceived patient expectations for antibiotics, and other situational factors.**

A&F interventions consist of proceeding through one or more cycles of establishing best practice criteria, measuring current practice, feeding back findings, implementing changes, and further monitoring of data [18]. All aspects of the cycle are important to facilitate *information* to *intention* and ultimately link that intention to the desired *behavior* [28]*.* Clinicians need to accept the data presented to them, understand how to interpret the data and the expected action to take as a result. Gaps or weaknesses in any of these components of the cycle will create an *intention-to-action* gap reducing the desired effect; in this case resulting in no reduction in inappropriate antibiotic prescribing [29].

**Strategies to optimize reach and engagement of an antibiotic audit and feedback intervention in primary care include; utilizing an opt-out approach to delivery of feedback reports, offering of continuing medical education credits, financial incentives, and facilitated peer group discussions.**

The starting point for A&F interventions is that they are important quality improvement initiatives. The poorest performing clinicians benefit the most from A&F, but are also least likely to volunteer [10]. For this reason we advocate that inclusion to receive A&F should be standard of practice and an opt-out approach be taken; where prescribers routinely receive feedback (unless opted out) and are not simply invited to volunteer. A&F should be integrated into routine professional quality improvement activities and electronic health records to maximize the potential impact [12, 13]. Other strategies and incentives that can be effective, depending on the local context, include offering of continuing medical education credits, accreditation and/or financial incentives for participation [30].

## Selecting feedback recipients

**All primary care prescribers, regardless of practice type or prescribing volume, should be included in antibiotic prescribing audit and feedback interventions.**

While there was general agreement that receiving A&F should follow an opt-out approach (statement 3), however, discussions during the MDP meetings revealed a lack of consensus whether A&F programs should preferentially include a subset of prescribers (eg; high or poor performing prescribers). MDP participants articulated that selecting appropriate prescribers to be involved in A&F is context dependent. As discussed above A&F is a quality improvement initiative that should be standard practice in all jurisdictions. All prescribers may benefit from their data with peer comparisons and/or comparison with standard of care, with opportunities to improve. The goal of antibiotic A&F at the population is to shift the mean towards lower inappropriate antibiotic use, and avoiding regression to the mean whereby lower prescribers increase their antibiotic use. Targeting only the highest prescribers, or lowest performing physicians, may avoid regression to the mean as well as be more cost effective [12, 13]. We recommend those initiating A&F in primary care to consider their local context and selected data metrics for selecting recipients for the proposed intervention recognizing there is uncertainty if there is benefit in focusing feedback to all prescribers.

## Data and indicator selection

**Feedback indicators for antibiotic prescribing in primary care should target reductions in antibiotic initiations, prolonged antibiotic duration, and/or unnecessary broad-spectrum antibiotics.**

As demonstrated by the CP-FIT model, designers of feedback interventions should consider importance, controllability, and relevance [18]. Data indicators for antibiotic A&F should therefore be linked to quality improvement, be under the control of the feedback recipient, and clearly link to specific actions that can be taken by the recipient [17]. Antibiotic initiations, durations, and overly broad-spectrum selection, relative to their peers or benchmarks, are all associated with unnecessary antibiotic-associated harms and antimicrobial resistance [31–34]. Furthermore, these represent three distinct prescribing behaviours that are not correlated to one another for an individual prescriber [35]. We recommend A&F designers select indicators by considering their data availability for each of these options and the desired prescribing behavior they wish to influence. We encourage A&F designers to focus on a clear, specific targeted indicator to minimize data overload. There is a risk that clinicians will focus on the indicators for which they are doing well if provided with multiple different indicators [15].

Total antibiotic use is highly correlated with unnecessary or inappropriate use [36], and in jurisdictions with antibiotic overuse, utilizing overall antibiotic prescriptions can be an effective and appropriate indicator to reduce unnecessary antibiotic prescribing [12, 13]. However, the specificity of the indicator will be more related to actionability (eg; antibiotics for viral infections, feedback on prolonged duration prescribing, or selection of broad-spectrum agents). Utilizing more specific metrics may be more likely to modify behaviour, but may be less impactful for population level effects on AMR. Examples of quality indicators for outpatient antibiotic prescribing in Europe have been previously defined and can help guide indicator selection [37].

**Antibiotic feedback reports in primary care should enable and support behaviour change by providing guidance and educational resources.**

Feedback intervention designers should consider how supporting resources and guidance use behavioural techniques to support practice change. Providing antibiotic guidelines or patient directed educational materials may not by itself significantly improve the effect of A&F [12, 13], however antibiotic A&F can be optimized by including behaviour change messaging alongside peer comparison data [38]. This can address CP-FIT variables ‘problem solving’ and ‘action planning’ to help feedback recipients identify the reasons and solutions for poor performance [18]. It is important to link A&F data to specific actions the prescriber can take [17]. A&F may be delivered as part of clinical decision support and the MDP panel encourages the use of reputable local clinical guidelines and educational resources [12, 13].

**The optimal data source for antibiotic audit and feedback in primary care is credible, valid, routinely collected, and comprehensive for the region; ideally containing prescription, diagnostic, and clinical data.**

The CP-FIT suggests that data collection and analysis for feedback should be automated, accurate, and accepted by recipients [18]. One of the most common criticisms from A&F recipients is that the data are not valid or do not accurately reflect the prescribers practice [39]. Programs should consider and attempt to address these factors upfront to support the credibility of the data and optimize the chance of success.

**Benchmarks or achievable targets for peer comparisons for antibiotic prescribing in primary care should be indicator-specific and based on national and/or local performance data of high performing peers.**

In the absence of being able to accurately define inappropriate antibiotic prescriptions within the data used for feedback, the optimal target, or benchmark, that should be used is controversial and depends on the indicator used, data availability, and the existence of local antibiotic prescribing guidelines. Previous interventions have had varied comparators. A study by Meeker *et al*. effectively compared physicians to their top decile of their peers for unnecessary antibiotic prescribing for viral infections [14]. Hallsworth et al. [13] compared high prescribing practices (top 20th percentile) to lower prescribing practices. Quality indicators have been proposed in Dutch primary care which include; the number of antibiotic prescriptions per 1000 registered patients, non-first line antibiotics, and the percentage of antibiotics for respiratory tract infections [40]. Given the lack of evidence defining optimal comparators, or benchmarks, JPIAMR-PAAN has recognized this topic as an important research priority. In general, comparators should be selected which reinforce the desired behavior change (i.e. reduce unnecessary or inappropriate antibiotic prescribing) [17]. We encourage selection of only a single comparator since using multiple comparators risks creating conflicting messages for recipients. For example, providing self-comparison showing decreasing trends over time as well as a peer comparison that has a lower prescribing rate may result in the recipient interpreting the data that they do not need to change based on their decreased trend. Designers should consider the desired behavior change and strategically select a simple, clear comparator that reinforces the project's goals.

## Feedback delivery

**Antibiotic audit and feedback in primary care should be displayed such that recipients can understand their performance and desired actions within seconds.**

Primary care clinicians are busy, manage a broad range of patients, and have multiple competing priorities for quality improvement. Designers of A&F interventions should minimize extraneous cognitive load for feedback recipients by prioritizing the relative importance of feedback contents and employ user-friendly designs [18]. Overly complex feedback can be misunderstood or ignored by the recipients [17]. Extraneous cognitive load can be minimized by reducing the number of metrics, decreasing the length of letters, and decluttering visual displays. A clear graphical interactive display facilitates interpretability of the feedback and has been demonstrated in an Australian intervention to reduce antibiotic use compared to letters without such a figure [41].

**Antibiotic audit and feedback reports in primary care should be repeated with updated data over time. The optimal frequency is not known but can depend on local factors such as data availability and seasonality of prescribing.**

A&F provided more than once is generally more effective [10]. Providing A&F repeatedly can encourage a feedback loop where a recipient can receive feedback, make changes in their practice, and then see whether the changes made have been effective [17]. Antibiotic A&F interventions have varied in frequency from once per year [12, 13], quarterly [15, 42], or monthly [14]. One trial of A&F to dental practices evaluated providing feedback 2 or 3 times per year with no differences between the groups [38]. There is not currently enough evidence to suggest whether providing feedback more than twice has a meaningful impact. Further evidence on the optimal frequency of feedback is needed but we encourage designers to include multiple instances of feedback of at least once per year [10]. Other considerations are the availability and delays in data as well as not overburdening recipients with frequent reports.

**Antibiotic feedback in primary care should be ideally delivered by multiple strategies including verbal, paper, and/or electronic means.**

The Cochrane review on A&F identified larger effect sizes when feedback is provided through both written and verbal communication [10]. The combination can enhance learning, retention, and engagement with the feedback reports. Providing an opportunity for recipients to engage with their feedback in more than one way including peer discussions may enhance the effectiveness of the intervention.

**Antibiotic feedback should be delivered to primary care prescribers from a respected authority figure or colleague.**

A common criticism of A&F by recipients is the credibility of the information provided [39]. CP-FIT acknowledges that source knowledge and skill is an important variable for feedback delivery. In order to facilitate practice change recipients must perceive the feedback content as credible, and the delivery person to have an appropriate level of knowledge [18]. The Cochrane review on A&F identified that feedback delivered by a supervisor or colleague was more effective than interventions delivered from other sources [10]. An example of this is A&F in the United Kingdom delivered from the Chief Medical Officer of Health [13].

**Individual-level antibiotic feedback should be delivered confidentially to primary care prescribers, and the opportunity for peer discussion should be provided and encouraged.**

Where possible, individual feedback, opposed to group or regional level feedback, should be provided to address specificity. The personalized nature of A&F facilitates the desired behaviour change and is less likely to be discounted by the recipients. Engaging with the feedback through social interaction is based on educational research on improved adult learning through social construction compared to passively received materials. Peer discussion and active delivery of feedback are both supported by CP-FIT to improve effectiveness of feedback interventions [18]. Further dialogue with peers can help to further enhance engagement in self-assessment for recipients and optimize the effectiveness of A&F interventions [43].

## Discussion

Antibiotic A&F for primary care clinicians is a potentially effective population level intervention to improve antibiotic use and combat rising AMR. However, the design and delivery of these interventions are critical to their success. The best practice statements found in this study provides 13 recommendations for designers of antibiotic primary care A&F interventions which builds on existing evidence, best practices for A&F in general [17], the CP-FIT [18], and expert consensus.

Best practice recommendations for A&F exist and this work builds on those recommendations to provide more specificity related to A&F on antibiotics in primary care. Table 1 outlines areas of agreement between our recommendations and existing literature on A&F in general, and highlights recommendations specific to antibiotic A&F in primary care.

Inappropriate antibiotic prescribing behaviour is complex and driven by habit, fear, time constraints, and perceived patient expectations. Rarely, is it related to a simple knowledge gap to be affected by education alone [44]. Multifaceted approaches to antibiotic prescribing in primary care in order to facilitate a change in prescribing are essential. A&F should be implemented alongside clinical decision support, point-of-care diagnostics, patient and prescriber education, and safety netting procedures. A&F is an important component of antimicrobial stewardship efforts which can address some of the barriers to change and has demonstrated effectiveness in reducing antibiotic use. However, the design and details may make the difference between effective and ineffective interventions [11].

We have included a toolkit to facilitate the application of these 13 best practice recommendations as well as examples of applying the toolkit to previously published trials (Additional file 1: B). This toolkit is also available on our website www.jpiamr-paan.org.

There are limitations to this guidance document. Not all statements will apply to all interventions depending on local context and data availability. There are important gaps in the evidence with some aspects more heavily influenced by previous experience and expert opinion. The modified Delphi process provides some rigour to these opinions and we highlighted within each statement areas of gaps in evidence which should be prioritized for future research. AMR is a global problem that impacts low and middle income countries disproportionately. However, the etiology of rising AMR, and actions required to combat them, have important differences compared to high income countries. These recommendations focus on A&F specifically for high income countries and may not be appropriate for low or middle-income countries.

We encourage antimicrobial stewardship and public health programs to utilize this guidance and the checklist provided in Additional file 1: B to consider these 13 components in an effort to optimize the impact and success of antibiotic A&F in primary care. Urgent action globally is needed to combat AMR through overuse of antibiotics. A&F should be a core component of national AMR action plans to reduce inappropriate antibiotic use in primary care.

## Supplementary Information

**Additional file 1:** Supplementary materials.

## Data Availability

All data supporting the findings of this study are available within the paper and its Additional file 1.
