# Supplementary material for: Best practice guidance for antibiotic audit and feedback interventions in primary care: a modified Delphi study from the Joint Programming Initiative on Antimicrobial resistance: Primary Care Antibiotic Audit and Feedback Network (JPIAMR-PAAN)
Source: Antimicrob Resist Infect Control. 2023 Jul 29;12:72. doi: 10.1186/s13756-023-01279-z (PMC10387210; doi:10.1186/s13756-023-01279-z)
Supplement: Supplementary file 1 — Additional file 1: Supplementary materials. [file 13756_2023_1279_MOESM1_ESM.docx]

**Supplementary Materials**

Supplementary Table S1: JPIAMR-PAAN Members

| Name | Country | Credentials and Primary Affiliations | Modified Delphi Process Participation |
| --- | --- | --- | --- |
| Anna Acampora | Italy | Researcher, Department of Epidemiology, Rome, Lazio Region | N/A |
| Sarah Alderson | UK | Associate Professor of Primary Care, Leeds Institute of Health Sciences, University of Leads | Survey 1A, 1B, 2, and 3; Feedback meeting #1 and #2 |
| Pablo Alonso Coello | Spain | Senior Researcher, Institute of Research of Hospital de la Santa Creu i Sant Pau | Survey 1A, 1B, and 2; Feedback meeting #2 |
| Attila Altiner | Germany | Professor, Medical Director, Department of General Practice and Health Services Research, Heidelberg University | N/A |
| Lars Bjerrum | Denmark | Professor, Section of General Practice, University of Copenhagen | Survey 1A, 1B, 2, and 3; Feedback meeting #1 and #2 |
| Jamie Brehaut | Canada | Senior Scientist, Ottawa Hospital Research Institute;  Professor, School of Epidemiology and Public Health, University of Ottawa | N/A |
| Benjamin Brown | UK | Senior Researcher, Division of Population health, Health Services Research and Primary Care, University of Manchester | Survey 1A, 1B, 2, and 3 |
| Heiner Bucher | Switzerland | Professor of Clinical Epidemiology, University of Basel; Director, Basel Institute for Clinical Epidemiology and Biostatistics, University Hospital Basel | Survey 1A and 3; Feedback meeting #2 |
| Chris Butler | UK | Professor, Nuffield Department of Primary Care Health Sciences, University of Oxford | N/A |
| Laura Cavazzuti | Italy | Researcher, Azienda Sanitaria Locale-IRCCS of Reggio Emilia | Survey 1A, 1B, 2, and 3 |
| Janet Clarkson | UK | Professor, Director, Dental Health Services Research Unit, University of Dundee;  Dental Dean, NHS Education for Scotland;  Professor of Dental Research, University of Manchester | Survey 1A, 1B, and 2; Feedback meeting #2 |
| Marina Davoli | Italy | Researcher, Department of Epidemiology, Rome, Lazio Region | Survey 1A |
| An De Sutter | Belgium | Professor, Department of Public Health and Primary Care, Ghent University | N/A |
| Mirko Di Martino | Italy | Researcher, Department of Epidemiology, Rome, Lazio Region | N/A |
| Eilidh Duncan | UK | Research Fellow, Health Services Research Unit, University of Aberdeen | Survey 1A, 1B, 2, and 3; Feedback meeting #1 and #2 |
| Nick Francis | UK | Professor, School of Primary Care, Population Sciences and Medical Education, University of Southampton | N/A |
| Roberto Grilli | Italy | Director, Health Services Research Evaluation and Policy Unit, Azienda Sanitaria Locale-IRCCS of Reggio Emilia | Survey 1A, 1B, 2, and 3 |
| Jeremy Grimshaw | Canada | Senior Scientist, Ottawa Hospital Research Institute;  Professor, Department of Medicine, University of Ottawa | Survey 1A; Feedback meeting #1 and #2 |
| Ronny Gunnarsson | Sweden | Professor, School of Public Health and Community Medicine, University of Gothenburg;  Scientist, Research, Education, Development, Education and Innovation, Primary Health Care, Region Västra Götaland | Survey 2 and 3 |
| Michael Hallsworth | USA | Managing Director, Behavioral Insights Team | N/A |
| Lars Hemkens | Switzerland | Senior Scientist, Department of Clinical Research, University of Basel | N/A |
| Sigurd Høye | Norway | Associate Professor, Department of General Practice, University of Oslo | Survey 1B and 3; Feedback meeting #2 |
| Noah Ivers | Canada | Scientist, Women's College Hospital;  Associate Professor, Department of Family & Community Medicine, University of Toronto | Survey 2 and 3; Feedback meeting #2 |
| Tasneem Khan | UK | Research Fellow, Leeds Institute of Health Sciences, University of Leeds | Survey 1B and 3 |
| Donna Lecky | UK | Head, Primary Care & Interventions Unit, UK Health Security Agency | Survey 1B and 2; Feedback meeting #1 |
| Morten Lindbæk | Norway | Professor, Senior Advisor, Department of General Practice, University of Oslo | Survey 1A, 1B, 2, and 3; Feedback meeting #1 and #2 |
| Jeff Linder | USA | Professor, Department of Medicine, Northwestern University | N/A |
| Paul Little | UK | Professor, Primary Care Research Centre, University of Southampton | Feedback meeting #1 |
| Carl Llor | Spain; Denmark | Scientist, University Institute in Primary Care Research Jordi Gol, Via Roma Health Centre;  Associate Professor, Department of Public Health, University of Southern Denmark | Survey 1A, 1B, 2, and 3; Feedback meeting #1 |
| Fabiano Lorencatto | UK | Research Lead, Centre for Behaviour Change University College London | Survey 1A |
| Denise O'Connor | Australia | Professor, School of Public Health and Preventive Medicine, Monash University | Survey 1B, 2, and 3 |
| Celine Pulcini | France | Professor, University of Lorraine and Nancy University Hospital | Survey 1A, 1B, 2, and 3; Feedback meeting #1 and #2 |
| Craig Ramsay | UK | Professor, Director of Health Services Research, University of Aberdeen | Survey 2; Feedback meeting #1 |
| Rosella Saulle | Italy | Researcher, Department of Epidemiology, Rome, Lazio Region | N/A |
| Kevin Schwartz | Canada | Scientist, Public Health Ontario;  Assistant Professor, Dalla Lana School of Public Health, University of Toronto | Survey 1A, 1B, 2, and 3; Feedback meeting #1 and #2 |
| Maia Simon | France | Research Fellow, University of Lorraine | Survey 1A, 1B, 2, and 3; Feedback meeting #1 |
| Pär-Daniel Sundvall | Sweden | Senior Lecturer, School of Public Health and Community Medicine, University of Gothenburg;  Scientist, Research, Education, Development, Education and Innovation, Primary Health Care, Region Västra Götaland | Survey 1A, 1B, 2, and 3; Feedback meeting #1 and #2 |
| Monica Taljaard | Canada | Senior Scientist, Ottawa Hospital Research Institute;  Professor, School of Epidemiology and Public Health, University of Ottawa | Survey 1A, 1B, and 3 |
| Pia Touboul Lundgren | France | Scientist, Nice University Hospital, Department of Public Health | Survey 3 |
| Akke Vellinga | Ireland | Professor, School of Public Health, Physiotherapy and Sports Science, University College Dublin | N/A |
| Jan Verbakel | Belgium | Associate Professor, Department of Public Health and Primary Care, KU Leuven | N/A |
| Theo Verheij | Netherlands | Professor, Department of General Practice and Nursing Science, University Medical Center Utrecht | Survey 1A, 1B, and 2; Feedback meeting #1 |

Supplementary Materials A: Surveys used in the Modified Delphi Process

SURVEY 1A

The objective of the present Modified Delphi Process (MDP) is to identify and develop best practice guidelines and toolkits for peer comparison audit and feedback of antibiotic prescribing in primary care settings in high-income countries. Panelists will include 30+ members of JPIAMR-PAAN from Australia, Europe, and North America. Panelists will undergo three rounds of questionnaires and ratings. This the first step in a process to develop tools and resources for those performing, or interested in initiating, audit and feedback for antibiotic prescribing in primary care. The following final statements based on your feedback will be refined and discussed at our upcoming meetings.

Survey 1A is Step 1 of 2 in the first round of the MDP. The objective of Survey 1A is to collect feedback and suggestions for a set of initial statements. Data collected in this survey will subsequently be reviewed and all panelists will have the opportunity to rate their agreement with each statement in Survey 1B.

Instructions to complete the survey

- Please consider each question and how you would answer them
- Please select all provided answer(s) that you agree with, or add more suggestions in the free text box below the question
- Please indicate whether or not you think the question should be included
- Please suggest edits to the wording of each question/answer
- Remember to consider the exact wording of both the question and the answer
- At the end of each section, you will have the opportunity to suggest additional questions not listed in the current survey
- This survey should take approximately 15-20 minutes to complete

GENERAL STATEMENTS ON AUDIT AND FEEDBACK

1. How should the audit and feedback intervention be framed in order to maximize the impact of the intervention?

Select ALL answers that you agree with. If you have other or alternative answers, please provide these in the free text box below each question.

• Quality improvement project

• Behavior change project

• Continuing Medical Education (CME) project

• Surveillance project

• Confidential feedback project

• Non punitive feedback project

• Other (please specify below)

Should question 1 be included?

• Yes

• No

2. What are the barriers to the development of audit and feedback interventions?

Select ALL answers that you agree with. If you have other or alternative answers, please provide these in the free text box below each question.

• Data availability (e.g. accessibility, cost)

• Data validity (e.g. data errors)

• Stakeholder buy-in from recipients of the feedback

• Other (please specify below)

Should question 2 be included?

• Yes

• No

3. Would you suggest other general statements on audit and feedback that should be included?

DESIRED ACTION

4. What population should be the focus for antibiotic prescribing audit and feedback interventions?

Select ALL answers that you agree with. If you have other or alternative answers, please provide these in the free text box below each question.

• High prescribers

• All prescribers

• Other (please specify below)

Should question 4 be included?

• Yes

• No

5. What type of prescribers should be included in the audit and feedback intervention?

Select ALL answers that you agree with. If you have other or alternative answers, please provide these in the free text box below each question.

• Physicians

• Pharmacists

• Dentists

• Nurses

• Other (please specify below)

Should question 5 be included?

• Yes

• No

6. What antibiotic prescribing indicators should be used to establish the desired goals and provide feedback on?

Select ALL answers that you agree with. If you have other or alternative answers, please provide these in the free text box below each question.

• Indicators used for feedback should be actionable and the desired behavior is under the control of the target

• Total antibiotic prescribing feedback should guide reduction in total use of antibiotics

• Inappropriate antibiotic prescribing feedback should guide reduction in total use of antibiotics

• Feedback on prolonged duration prescribing should guide shorter duration use

• Feedback on broad spectrum prescribing should guide narrow spectrum agent use

• Other (please specify below)

Should question 6 be included?

• Yes

• No

7. Which actions should be used to improve participation in the audit and feedback intervention?

Select ALL answers that you agree with. If you have other or alternative answers, please provide these in the free text box below each question.

• Participation in an audit and feedback activity should give continuing medical education credits

• Participation in an audit and feedback activity should be compulsory for prescribers

• Participation in an audit and feedback activity should be compensated

• Other (please specify below)

Should question 7 be included?

• Yes

• No

8. What other questions or statements related to “desired action” should be included?

DESIRED DATA

9. What is the ideal data source(s) for feedback?

Select ALL answers that you agree with. If you have other or alternative answers, please provide these in the free text box below each question.

• Administrative data

• Pharmacy claims data

• Electronic medical record/chart data

• Patient reported data

• Other (please specify below)

Should question 9 be included?

• Yes

• No

10. What indicators should be provided for feedback?

Select ALL answers that you agree with. If you have other or alternative answers, please provide these in the free text box below each question.

• Inappropriate or unnecessary antibiotic use is the preferred indicator.

• Proxy indicators can be used and should be aligned with the preferred indicators (For example: Total antibiotic use, although assumes some are inappropriate, cannot directly give feedback on inappropriate)

• One indicator should include proportion of broad-spectrum antibiotics on a predefined list, such as quinolones and macrolides

• On indicator should include duration (i.e.; proportion of prolonged duration prescriptions)

• Other (please specify below)

Should question 10 be included?

• Yes

• No

11. What are the preferred measures of antibiotic prescribing?

Select ALL answers that you agree with. If you have other or alternative answers, please provide these in the free text box below each question.

• Defined Daily Doses (DDDs)

• Number of antibiotic prescriptions

• Antibiotic prescription rate per consultation

• Antibiotic prescription rate per population or listed patients

• Other (please specify below)

Should question 11 be included?

• Yes

• No

12. What benchmarks are preferred for comparison?

Select ALL answers that you agree with. If you have other or alternative answers, please provide these in the free text box below each question.

• The median (total antibiotic use) should be used as the benchmark

• The benchmark should be based on the prescribing achieved by some well-performing peers

• The benchmark should be based on the prescribing achieved by similar peers

• The benchmark should be based on desired reduction in antibiotic prescribing in the population

• Other (please specify below)

Should question 12 be included?

• Yes

• No

13. What other questions or statements related to “desired data” should be included?

FEEDBACK DISPLAY

14. What are the preferred displays of feedback?

Select ALL answers that you agree with. If you have other or alternative answers, please provide these in the free text box below each question.

Examples of feedback display are available here: https://docs.google.com/document/d/1N3Q2mnHS-hhkx8CQz5-04T4TDwUEh8gWELLiMH-xt8A/edit?usp=sharing

• A central figure is an important component of the feedback

• Multiple figures for all available indicators

• Key indicators should be displayed for comparison with other participants in a peer group

• Include general information on antimicrobial resistance

• Include links to guidelines

• Other (please specify below)

Should question 14 be included?

• Yes

• No

15. What is the preferred frequency for feedback?

Select ALL answers that you agree with. If you have other or alternative answers, please provide these in the free text box below each question.

• Immediately (e.g. via an electronic dashboard)

• Monthly

• Quarterly

• Semi-annually

• Annually

• Other (please specify below)

Should question 15 be included?

• Yes

• No

16. What other questions or statements related to “feedback display” should be included?

AUDIT AND FEEDBACK DELIVERY

17. What is the preferred mechanism of providing feedback?

Select ALL answers that you agree with. If you have other or alternative answers, please provide these in the free text box below each question.

• Electronic dashboard

• Electronic mail

• Paper mail

• In a peer group setting

• Combinations

• Other (please specify below)

Should question 17 be included?

• Yes

• No

18. Who should feedback be provided from?

Select ALL answers that you agree with. If you have other or alternative answers, please provide these in the free text box below each question.

• A respected colleague

• Authority figure (e.g. government officials)

• Non-government officials (e.g. physician colleges)

• Other (please specify below)

Should question 18 be included?

• Yes

• No

19. Who should the feedback be delivered to?

Select ALL answers that you agree with. If you have other or alternative answers, please provide these in the free text box below each question.

• Team

• Individual

• Other (please specify below)

Should question 19 be included?

• Yes

• No

20. What other questions or statements related to audit and feedback delivery should be included?

SURVEY 1B

Survey 1B is Step 2 of 2 in the first round of the Modified Delphi Process (MDP). The objective of Survey 1B is to allow panelists to rate their level of agreement with the initial set of statements developed with feedback collected from Survey 1A. Each statement will be rated on the Five-point Likert Scale (1-Strongly Disagree to 5-Strongly Agree). Additional details and commentary will follow each statement in the final document. We welcome comments in the free text which will be incorporated into this commentary.

Instructions to complete the survey:

- Please consider each statement below in the context of developing an audit and feedback intervention for antibiotic prescribing in primary care.
- Please rate your level of agreement with each statement and its wording using the Five-Point Likert Scale, or indicate that this statement should not be included.
- Use the free text box at the end of each statement to leave comments, feedback, or suggestions for the statement.
- This survey should take approximately 10-15 minutes to complete.

GENERAL STATEMENTS ON AUDIT AND FEEDBACK

1. In order to maximize the impact of audit and feedback interventions, they should be framed as quality improvement projects within a supportive environment.

2. When developing audit and feedback interventions for primary care antibiotic prescribing, barriers that should be considered and addressed include data availability and validity, as well as engaging the interest of prescribers receiving feedback.

STATEMENTS ON DESIRED ACTION

3a) All prescribers should be included in antibiotic prescribing audit and feedback interventions.

3b) High volume, or poor performing prescribers, should NOT be the focus of antibiotic prescribing audit and feedback interventions.

4a) All types of prescribers should be included in antibiotic audit and feedback interventions, including primarily physicians, as the most frequent prescribers, but also pharmacists, dentists, and nurses.

4b) In long term care or nursing home facilities, nursing and support staff (such as nursing assistants or personal support workers) should be included in facility level audit and feedback interventions.

5a) Indicators used for feedback should be actionable and the desired behavior to be changed should be under the control of the target. As such, indicators should be framed in terms of actions or behavior changes rather than outcomes.

5b) Indicators for feedback directly measuring inappropriate antibiotic prescribing should be used to target reductions in unnecessary antibiotic initiations, prolonged antibiotic durations, and/or unnecessarily broad spectrum antibiotics.

- The following publications are examples of inappropriate antibiotic indicators;
  - initiation: <https://pubmed.ncbi.nlm.nih.gov/26864410/>
  - prolonged duration: <https://pubmed.ncbi.nlm.nih.gov/33754632/>
  - selection: <https://pubmed.ncbi.nlm.nih.gov/28397171/>

5c) Total antibiotic use, as a proxy for overuse, should NOT be used if other indicators are available.

- Example: <https://pubmed.ncbi.nlm.nih.gov/34228086/>

6. As a way to improve participation in the audit and feedback intervention, continuing medical education credits should be provided to prescribers.

7. Receiving antibiotic audit and feedback reports should ideally be compulsory, without opportunity to opt out, or structured as part of an accreditation process.

8. Resources should be provided to prescribers to enable and support changes to prescribing behavior based on the feedback report.

STATEMENTS ON DESIRED DATA

9. The optimal data source for antibiotic audit and feedback is routinely collected, comprehensive for the region, and valid; containing prescription, diagnostic, and clinical data.

10a) The preferred benchmark for peer comparison should be to high-performing peers with similar practice settings.

10b) The median should NOT be used as the comparator.

STATEMENTS ON FEEDBACK DISPLAY

11a) The preferred display of feedback should incorporate input from behavioral science specialists, and include key indicators displayed graphically for comparison with other participants in a peer group.

11b) In addition, links to guidelines and general information on antimicrobial resistance are also key components of feedback to link intention to action.

12. The preferred frequency for feedback is quarterly, if data are available and there is adequate volume of prescribing for reliable estimates. Feedback may be provided less frequently, but may be less impactful.

STATEMENTS ON FEEDBACK DELIVERY

13. The ideal feedback delivery includes a combination of mechanisms, including electronic dashboards, email, and/or verbal feedback.

14. Feedback should ideally be delivered by scientific non-government officials or a respected colleague.

15a) Feedback should be delivered confidentially to the individual prescribers.

15b) Team-based feedback for collective review in a peer group is recommended for group practice settings to facilitate discussion.

ADDITIONAL QUESTIONS

16. Which co-interventions would you recommend to be used alongside audit and feedback to support prescribers to change practice?

17. Do you have any general comments for the Delphi process or suggestions for discussion at our first meeting (April 21, 2022)?

SURVEY 2

Survey 2 is Step 2 of 2 in the second round of the Modified Delphi Process (MDP), following our first virtual Delphi meeting. The objective of Survey 2 is to allow panelists to rate their level of agreement and provide comments and suggestions for the set of statements developed with feedback collected from the first round. Each statement will be rated on the Five-point Likert Scale (1-Strongly Disagree to 5-Strongly Agree).

Instructions

- Please consider each statement below in the context of developing an audit and feedback intervention for antibiotic prescribing in primary care
- Please rate your level of agreement with each statement and its wording using the Five-Point Likert Scale.
- Use the free text box at the end of each statement to leave comments, feedback, or suggestions for the statement.
- Note that a detailed rationale will be included for each statement in the final publication to provide context and other considerations.
- This survey should take approximately 15 minutes to complete

GENERAL CONSIDERATIONS FOR ANTIBIOTIC AUDIT AND FEEDBACK IN PRIMARY CARE

1. In order to maximize the impact of the audit and feedback interventions, they should be framed as quality improvement projects within a supportive environment.

2. When developing audit and feedback interventions, barriers that should be considered and addressed include data availability and validity, as well as engaging the interest of prescribers receiving feedback.

3. When developing audit and feedback interventions, consideration should be given on strategies to optimize engagement by the prescribers. Engagement can be enhanced through passive (i.e. non-voluntary) delivery of feedback reports, as well as adopting incentives which can include continuing medical education credits, peer group discussion, and/or financial incentives.

SELECTING PRESCRIBERS FOR ANTIBIOTIC AUDIT AND FEEDBACK IN PRIMARY CARE

4. All prescribers, regardless of practice type or prescribing volume, should ideally be included in antibiotic prescribing audit and feedback interventions. A focus on high, or poor performing, prescribers can be used depending on the data and metrics available for use.

5. Physicians are the priority group as the most frequent antibiotic prescribers, but pharmacists, dentists, and nurses should be considered as feedback recipients depending on local context. In long term care or nursing home settings, a multifaceted intervention is necessary to provide actionable audit and feedback reports for prescribers, while also engaging support staff (such as nurses and personal support workers) with antimicrobial stewardship co-interventions.

DATA AND INDICATOR SELECTION FOR AUDIT AND FEEDBACK IN PRIMARY CARE

6. Feedback indicators for antibiotic prescribing should target reductions in unnecessary antibiotic initiations, prolonged antibiotic duration, and/or unnecessary broad-spectrum antibiotics. Depending on data availability and estimated regional antibiotic overuse, total antibiotic use can be used as a proxy indicator. Denominators, such as number of patients or per indication, should be included for each prescribing indicator.

7. Guidance and education should be provided to prescribers to enable and support changes to prescribing behavior based on the feedback report.

8. The optimal data source for antibiotic audit and feedback is routinely collected, comprehensive for the region, and valid; containing prescription, diagnostic, and clinical data. Non-optimal data sources can also be effectively utilized to improve prescribing.

9. Benchmarks or achievable targets for peer comparison should be indicator specific and based on national and/or local performance data of high performing peers. Benchmarks or achievable targets can also vary based on the baseline performance of the feedback recipient.

ANTIBIOTIC AUDIT AND FEEDBACK DELIVERY IN PRIMARY CARE

10. Components of effective feedback display can include actionable behavior change messaging, and graphical peer comparisons, which minimizes extraneous cognitive load for recipients.

11. The preferred frequency for feedback is semiannually.

12. The ideal feedback delivery includes a combination of mechanisms, including electronic dashboards, email, and/or verbal feedback.

13. Feedback should ideally be delivered by scientific non-government officials or a respected colleague.

14. Individual-level feedback should be delivered confidentially to prescribers, but the opportunity for peer discussion should be provided and encouraged.

SURVEY 3

Survey 3 is the final step of the Modified Delphi Process (MDP). The objective of Survey 3 is to allow panelists to rate their level of agreement with the set of statements developed with feedback collected from the first two rounds. Each statement will be rated on the Five-point Likert Scale (1-Strongly Disagree to 5-Strongly Agree).

Instructions

- Please consider each statement below in the context of developing an audit and feedback intervention for antibiotic prescribing in primary care
- Please rate your level of agreement with each statement and its wording using the Five-Point Likert Scale.
- Note that a detailed rationale will be included for each statement in the final publication to provide context and other considerations.
- This survey should take approximately 10 minutes to complete

GENERAL CONSIDERATIONS FOR ANTIBIOTIC AUDIT AND FEEDBACK IN PRIMARY CARE

1. Antibiotic audit and feedback interventions in primary care should be framed as quality improvement projects within a supportive environment.

2. Prior to initiating an antibiotic audit and feedback intervention in primary care consider potential barriers to success such as local data availability, data validity, expected engagement of feedback recipients, perceived patient expectations for antibiotics, and other situational factors.

3. Strategies to optimize reach and engagement of an antibiotic audit and feedback intervention in primary care include; utilizing an opt-out approach to delivery of feedback reports, offering of continuing medical education credits, financial incentives, and facilitated peer group discussions.

SELECTING PRESCRIBERS FOR ANTIBIOTIC AUDIT AND FEEDBACK IN PRIMARY CARE

4. All primary care prescribers, regardless of practice type or prescribing volume, should be included in antibiotic prescribing audit and feedback interventions.

DATA AND INDICATOR SELECTION FOR AUDIT AND FEEDBACK IN PRIMARY CARE

5. Feedback indicators for antibiotic prescribing in primary care should target reductions in unnecessary antibiotic initiations, prolonged antibiotic duration, and/or unnecessary broad-spectrum antibiotics.

6. Antibiotic feedback reports in primary care should enable and support behaviour change by providing guidance and educational resources.

7. The optimal data source for antibiotic audit and feedback in primary care is credible, valid, routinely collected, and comprehensive for the region; ideally containing prescription, diagnostic, and clinical data.

8. Benchmarks or achievable targets for peer comparisons for antibiotic prescribing in primary care should be indicator specific and based on national and/or local performance data of high performing peers.

ANTIBIOTIC AUDIT AND FEEDBACK DELIVERY IN PRIMARY CARE

9. Antibiotic audit and feedback in primary care should be displayed such that recipients can understand their performance and desired actions within seconds.

10. Antibiotic audit and feedback reports in primary care should be repeated. The optimal frequency is not known but can depend on local factors such as data availability and seasonality of prescribing.

11. Antibiotic feedback in primary care should be ideally delivered by multiple strategies including verbal, paper, and/or electronic means.

12. Antibiotic feedback should be delivered to primary care prescribers by a respected authority figure or colleague.

13. Individual-level antibiotic feedback should be delivered confidentially to primary care prescribers, and the opportunity for peer discussion should be provided and encouraged.

Supplementary Materials B: Toolkit for designing and evaluating an A&F intervention in primary care for antibiotic prescribing.

| Researchers can work through this checklist to consider the 13 recommendations for primary care antibiotic A&F interventions from JPIAMR-PAAN. For each statement, consider if it has been, or can be, incorporated into your A&F intervention. Briefly consider how it has been incorporated, or if not, whether and when it can be addressed. It may be helpful to consider the reasons or limitations why cannot be incorporated to proactively support the intervention credibility. | |
| --- | --- |
| **GENERAL CONSIDERATIONS** | |
| **1. Antibiotic audit and feedback interventions in primary care should be framed as quality improvement projects within a supportive environment** | |
| **Yes** | How has this been incorporated? |
| **Partially** | How can it be incorporated? |
| **Not at all** | Why can it not be incorporated? |
| **2. Prior to initiating an antibiotic audit and feedback intervention in primary care consider potential barriers to success such as local data availability, data validity, expected engagement of feedback recipients, perceived patient expectations for antibiotics, and other situational factors** | |
| **Yes** | How has this been incorporated? |
| **Partially** | How can it be incorporated? |
| **Not at all** | Why can it not be incorporated? |
| **3. Strategies to optimize reach and engagement of an antibiotic audit and feedback intervention in primary care include; utilizing an opt-out approach to delivery of feedback reports, offering of continuing medical education credits, financial incentives, and facilitated peer group discussions** | |
| **Yes** | How has this been incorporated? |
| **Partially** | How can it be incorporated? |
| **Not at all** | Why can it not be incorporated? |
| **SELECTING PRESCRIBERS** | |
| **4. All primary care prescribers, regardless of practice type or prescribing volume, should be included in antibiotic prescribing audit and feedback interventions** | |
| **Yes** | How has this been incorporated? |
| **Partially** | How can it be incorporated? |
| **Not at all** | Why can it not be incorporated? |
| **DATA AND INDICATOR SELECTION** | |
| **5. Feedback indicators for antibiotic prescribing in primary care should target reductions in antibiotic initiations, prolonged antibiotic duration, and/or unnecessary broad-spectrum antibiotics** | |
| **Yes** | How has this been incorporated? |
| **Partially** | How can it be incorporated? |
| **Not at all** | Why can it not be incorporated? |
| **6. Antibiotic feedback reports in primary care should enable and support behaviour change by providing guidance and educational resources** | |
| **Yes** | How has this been incorporated? |
| **Partially** | How can it be incorporated? |
| **Not at all** | Why can it not be incorporated? |
| **7. The optimal data source for antibiotic audit and feedback in primary care is credible, valid, routinely collected, and comprehensive for the region; ideally containing prescription, diagnostic, and clinical data** | |
| **Yes** | How has this been incorporated? |
| **Partially** | How can it be incorporated? |
| **Not at all** | Why can it not be incorporated? |
| **8. Benchmarks or achievable targets for peer comparisons for antibiotic prescribing in primary care should be indicator specific and based on national and/or local performance data of high performing peers** | |
| **Yes** | How has this been incorporated? |
| **Partially** | How can it be incorporated? |
| **Not at all** | Why can it not be incorporated? |
| **FEEDBACK DELIVERY** | |
| **9. Antibiotic audit and feedback in primary care should be displayed such that recipients can understand their performance and desired actions within seconds** | |
| **Yes** | How has this been incorporated? |
| **Partially** | How can it be incorporated? |
| **Not at all** | Why can it not be incorporated? |
| **10. Antibiotic audit and feedback reports in primary care should be repeated. The optimal frequency is not known but can depend on local factors such as data availability and seasonality of prescribing** | |
| **Yes** | How has this been incorporated? |
| **Partially** | How can it be incorporated? |
| **Not at all** | Why can it not be incorporated? |
| **11. Antibiotic feedback in primary care should be ideally delivered by multiple strategies including verbal, paper, and/or electronic means** | |
| **Yes** | How has this been incorporated? |
| **Partially** | How can it be incorporated? |
| **Not at all** | Why can it not be incorporated? |
| **12. Antibiotic feedback should be delivered to primary care prescribers by a respected authority figure or colleague** | |
| **Yes** | How has this been incorporated? |
| **Partially** | How can it be incorporated? |
| **Not at all** | Why can it not be incorporated? |
| **13. Individual-level antibiotic feedback should be delivered confidentially to primary care prescribers, and the opportunity for peer discussion should be provided and encouraged** | |
| **Yes** | How has this been incorporated? |
| **Partially** | How can it be incorporated? |
| **Not at all** | Why can it not be incorporated? |

Below we have used previously published studies as an example of applying this toolkit in evaluating primary care antibiotic A&F interventions. The first was a study in Sweden by Hemkens et al. 2017.^15^ This study did not demonstrate an overall significant change in antibiotics. Eight, out of 13, recommendations were followed, 2 partially, and 3 not at all.

| **GENERAL CONSIDERATIONS** | |
| --- | --- |
| **1. Antibiotic audit and feedback interventions in primary care should be framed as quality improvement projects within a supportive environment** | |
| **Yes** | How has this been incorporated?  Along with the mailed feedback report (intervention), further information on the study were provided; including basic details on the rationale, privacy, and data protection issues. The intervention was described as a quality improvement program. |
| **Partially** | How can it be incorporated? |
| **Not at all** | Why can it not be incorporated? |
| **2. Prior to initiating an antibiotic audit and feedback intervention in primary care consider potential barriers to success such as local data availability, data validity, expected engagement of feedback recipients, perceived patient expectations for antibiotics, and other situational factors** | |
| **Yes** | How has this been incorporated?  Routine administrative (drug prescription and health care service) claims data were available and used for recruitment, intervention, and main outcome measurement. Participant engagement was promoted through multiple ways to access and interact with the feedback (e.g. individual access code to study website, evidence-based guidelines). |
| **Partially** | How can it be incorporated? |
| **Not at all** | Why can it not be incorporated? |
| **3. Strategies to optimize reach and engagement of an antibiotic audit and feedback intervention in primary care include; utilizing an opt-out approach to delivery of feedback reports, offering of continuing medical education credits, financial incentives, and facilitated peer group discussions** | |
| **Yes** | How has this been incorporated?  An opt-out approach was utilized as no informed consent was obtained to mail out the feedback reports; rather a response postcard for opting-out was provided. |
| **Partially** | How can it be incorporated? |
| **Not at all** | Why can it not be incorporated? |
| **SELECTING PRESCRIBERS** | |
| **4. All primary care prescribers, regardless of practice type or prescribing volume, should be included in antibiotic prescribing audit and feedback interventions** | |
| **Yes** | How has this been incorporated?  Selection criteria for physicians did not include practice type; however physicians with fewer than 100 patients per year were excluded. |
| **Partially** | How can it be incorporated? |
| **Not at all** | Why can it not be incorporated? |
| **DATA AND INDICATOR SELECTION** | |
| **5. Feedback indicators for antibiotic prescribing in primary care should target reductions in antibiotic initiations, prolonged antibiotic duration, and/or unnecessary broad-spectrum antibiotics** | |
| **Yes** | How has this been incorporated?  However, the primary outcome was the prescribed DDD of any type of antibiotics to any patient per 100 consultations. DDDs may be harder for the average physician to interpret and associate with behaviour change. Secondary prescribing metrics were provided by specific antibiotic type. |
| **Partially** | How can it be incorporated? |
| **Not at all** | Why can it not be incorporated? |
| **6. Antibiotic feedback reports in primary care should enable and support behaviour change by providing guidance and educational resources** | |
| **Yes** | How has this been incorporated?  Access to website provided, where additional details (on prescriptions per age group or sex or antibiotic type) and answers to frequently asked questions (on antibiotic use) were offered to participants. In addition, guidelines for optimized antibiotic use were mailed to participants along with the first feedback report. |
| **Partially** | How can it be incorporated? |
| **Not at all** | Why can it not be incorporated? |
| **7. The optimal data source for antibiotic audit and feedback in primary care is credible, valid, routinely collected, and comprehensive for the region; ideally containing prescription, diagnostic, and clinical data** | |
| **Yes** | How has this been incorporated?  Routinely collected prescription claims data were used for the intervention, which covers 64% of the Swiss population. |
| **Partially** | How can it be incorporated? |
| **Not at all** | Why can it not be incorporated? |
| **8. Benchmarks or achievable targets for peer comparisons for antibiotic prescribing in primary care should be indicator specific and based on national and/or local performance data of high performing peers** | |
| **Yes** | How has this been incorporated? |
| **Partially** | How can it be incorporated?  The intervention feedback report compared individual physicians with the adjusted average in peer physicians, rather than high performing peers. |
| **Not at all** | Why can it not be incorporated? |
| **DELIVERY** | |
| **9. Antibiotic audit and feedback in primary care should be displayed such that recipients can understand their performance and desired actions within seconds** | |
| **Yes** | How has this been incorporated? |
| **Partially** | How can it be incorporated? |
| **Not at all** | Why can it not be incorporated?  The feedback report was single-paged, with graphical displays that catches the eye and minimal text was utilized. However, a large amount of data provided in the dashboard h did not direct the recipients’ attention to the social comparison component. |
| **10. Antibiotic audit and feedback reports in primary care should be repeated. The optimal frequency is not known but can depend on local factors such as data availability and seasonality of prescribing** | |
| **Yes** | How has this been incorporated?  Given the limitation of administrative claims data being the time lag between prescription and reimbursement dates, quarterly feedback seemed appropriate for the region. |
| **Partially** | How can it be incorporated? |
| **Not at all** | Why can it not be incorporated? |
| **11. Antibiotic feedback in primary care should be ideally delivered by multiple strategies including verbal, paper, and/or electronic means** | |
| **Yes** | How has this been incorporated? |
| **Partially** | How can it be incorporated?  The intervention feedback report was only delivered by mail. Although an access code to the website was provided, the code was not delivered electronically. Following the first mailing, those who accessed the website should be asked to provide their email address so future reports may be emailed. |
| **Not at all** | Why can it not be incorporated? |
| **12. Antibiotic feedback should be delivered to primary care prescribers by a respected authority figure or colleague** | |
| **Yes** | How has this been incorporated? |
| **Partially** | How can it be incorporated? |
| **Not at all** | Why can it not be incorporated?  Physicians who received a feedback report were aware of the study and the validity of the data presented; however it was not delivered/signed by an authority figure. |
| **13. Individual-level antibiotic feedback should be delivered confidentially to primary care prescribers, and the opportunity for peer discussion should be provided and encouraged** | |
| **Yes** | How has this been incorporated? |
| **Partially** | How can it be incorporated? |
| **Not at all** | Why can it not be incorporated?  Peer discussion was not provided or explicitly encouraged as part of this intervention. However given the size and scope of the study, coordination of peer discussion would likely be resource-intensive and not feasible. |

Below we have used previously published studies as an example of applying this toolkit in evaluating primary care antibiotic A&F interventions. The second was a study in the United Kingdom by Hallsworth et al. 2016.^13^ This study did demonstrate an overall significant change in antibiotics. Nine, out of 13, recommendations were followed, 3 partially, and 1 not at all.

| **GENERAL CONSIDERATIONS** | |
| --- | --- |
| **1. Antibiotic audit and feedback interventions in primary care should be framed as quality improvement projects within a supportive environment** | |
| **Yes** | How has this been incorporated?  The main letter in the intervention did not suggest any punitive actions should participants fail to make changes. Rather the letter provided specific and feasible actions that participants could do to reduce unnecessary prescribing. |
| **Partially** | How can it be incorporated? |
| **Not at all** | Why can it not be incorporated? |
| **2. Prior to initiating an antibiotic audit and feedback intervention in primary care consider potential barriers to success such as local data availability, data validity, expected engagement of feedback recipients, perceived patient expectations for antibiotics, and other situational factors** | |
| **Yes** | How has this been incorporated?  The main intervention letter was addressed by a high-profile figure, which increased the credibility of the data. The patient-focused intervention following the letter directly address pressures from patients and aimed to promote more desirable attitude from patients. |
| **Partially** | How can it be incorporated? |
| **Not at all** | Why can it not be incorporated? |
| **3. Strategies to optimize reach and engagement of an antibiotic audit and feedback intervention in primary care include; utilizing an opt-out approach to delivery of feedback reports, offering of continuing medical education credits, financial incentives, and facilitated peer group discussions** | |
| **Yes** | How has this been incorporated?  This intervention utilized an opt-out approach. The letter encouraged discussion amongst peer prescribers as the prescribing data was recorded at a practice level. |
| **Partially** | How can it be incorporated? |
| **Not at all** | Why can it not be incorporated? |
| **SELECTING PRESCRIBERS** | |
| **4. All primary care prescribers, regardless of practice type or prescribing volume, should be included in antibiotic prescribing audit and feedback interventions** | |
| **Yes** | How has this been incorporated? |
| **Partially** | How can it be incorporated?  This intervention was limited to all high prescribing practices |
| **Not at all** | Why can it not be incorporated? |
| **DATA AND INDICATOR SELECTION** | |
| **5. Feedback indicators for antibiotic prescribing in primary care should target reductions in antibiotic initiations, prolonged antibiotic duration, and/or unnecessary broad-spectrum antibiotics** | |
| **Yes** | How has this been incorporated?  Total antibiotic prescribing targeted reductions in antibiotic initiations |
| **Partially** | How can it be incorporated? |
| **Not at all** | Why can it not be incorporated? |
| **6. Antibiotic feedback reports in primary care should enable and support behaviour change by providing guidance and educational resources** | |
| **Yes** | How has this been incorporated?  Additional resources were provided to physicians (e.g. TARGET leaflet to promote patient self-care) and patients (e.g. educational posters and letters). |
| **Partially** | How can it be incorporated? |
| **Not at all** | Why can it not be incorporated? |
| **7. The optimal data source for antibiotic audit and feedback in primary care is credible, valid, routinely collected, and comprehensive for the region; ideally containing prescription, diagnostic, and clinical data** | |
| **Yes** | How has this been incorporated?  Prescribing data came from datasets that were publicly available. |
| **Partially** | How can it be incorporated? |
| **Not at all** | Why can it not be incorporated? |
| **8. Benchmarks or achievable targets for peer comparisons for antibiotic prescribing in primary care should be indicator specific and based on national and/or local performance data of high performing peers** | |
| **Yes** | How has this been incorporated? |
| **Partially** | How can it be incorporated?  Feedback provided in the letter was non-specific (only indicated that the 80% of practices in the area prescribe fewer antibiotics per patient). As such, no specific benchmark or achievable target was available for direct comparison. However, prescribers were compared to their lower prescribing peers. |
| **Not at all** | Why can it not be incorporated? |
| **DELIVERY** | |
| **9. Antibiotic audit and feedback in primary care should be displayed such that recipients can understand their performance and desired actions within seconds** | |
| **Yes** | How has this been incorporated?  The main message (performance indicator) in the letter was easy to understand, with three clear and concise actionable behaviors to improve prescribing practices. |
| **Partially** | How can it be incorporated? |
| **Not at all** | Why can it not be incorporated? |
| **10. Antibiotic audit and feedback reports in primary care should be repeated. The optimal frequency is not known but can depend on local factors such as data availability and seasonality of prescribing** | |
| **Yes** | How has this been incorporated? |
| **Partially** | How can it be incorporated?  The main feedback letter was only delivered once in the trial (but subsequently delivered annually in the United Kingdom). |
| **Not at all** | Why can it not be incorporated? |
| **11. Antibiotic feedback in primary care should be ideally delivered by multiple strategies including verbal, paper, and/or electronic means** | |
| **Yes** | How has this been incorporated? |
| **Partially** | How can it be incorporated? |
| **Not at all** | Why can it not be incorporated?  Only one method of delivery (postal mail) was utilized by the intervention. |
| **12. Antibiotic feedback should be delivered to primary care prescribers by a respected authority figure or colleague** | |
| **Yes** | How has this been incorporated?  The feedback letter was signed off by the Chief Medical Officer. |
| **Partially** | How can it be incorporated? |
| **Not at all** | Why can it not be incorporated? |
| **13. Individual-level antibiotic feedback should be delivered confidentially to primary care prescribers, and the opportunity for peer discussion should be provided and encouraged** | |
| **Yes** | How has this been incorporated?  Peer discussion was encouraged as one of the three suggested actions to reduce prescribing. |
| **Partially** | How can it be incorporated? |
| **Not at all** | Why can it not be incorporated? |
